# Signaling pathway screening platforms are an efficient approach to identify therapeutic targets in cancers that lack known driver mutations: a case report for a cancer of unknown primary origin

**DOI:** 10.1038/s41525-018-0055-6

**Published:** 2018-06-20

**Authors:** Pedro Torres-Ayuso, Sudhakar Sahoo, Garry Ashton, Elvira An, Nicole Simms, Melanie Galvin, Hui Sun Leong, Kristopher K Frese, Kathryn Simpson, Natalie Cook, Andrew Hughes, Crispin J Miller, Richard Marais, Caroline Dive, Matthew G Krebs, John Brognard

**Affiliations:** 1 Signalling Networks in Cancer Group, Cancer Research UK, Manchester Institute, University of Manchester, Manchester, M20 4BX UK; 2 Signaling Networks in Cancer Section, Laboratory of Cell and Developmental Signaling, Center for Cancer Research, National Cancer Institute, Frederick, MD 21702 USA; 3 Computational Biology Support Team, Cancer Research UK, Manchester Institute, University of Manchester, Manchester, M20 4BX UK; 4 Histology, Cancer Research UK Manchester Institute, The University of Manchester, Manchester, M20 4BX UK; 5 Clinical and Experimental Pharmacology Group; Cancer Research UK Manchester Institute, The University of Manchester, Manchester, M20 4BX UK; 6 Division of Molecular and Clinical Cancer Sciences, Faculty of Biology, Medicine and Health, The University of Manchester and Experimental Cancer Medicine Team, The Christie NHS Foundation Trust, Manchester, M20 4BX UK; 7 Molecular Oncology Group, Cancer Research UK Manchester Institute, The University of Manchester, Manchester, M20 4BX UK; 8 Cancer Research UK Manchester Experimental Cancer Medicines Centre, The University of Manchester, Manchester, M20 4BX UK

## Abstract

Precision medicine aims to tailor cancer therapies to target specific tumor-promoting aberrations. For tumors that lack actionable drivers, which occurs frequently in the clinic, extensive molecular characterization and pre-clinical drug efficacy studies will be required. A cell line maintained at low passage and a patient- derived xenograft model (PDX) were generated using a fresh biopsy from a patient with a poorly-differentiated neuroendocrine tumor of unknown primary origin. Next-generation sequencing, high throughput signaling network analysis, and drug efficacy trials were then conducted to identify actionable targets for therapeutic intervention. No actionable mutations were identified after whole exome sequencing of the patient’s DNA. However, whole genome sequencing revealed amplification of the 3q and 5p chromosomal arms, that include the *PIK3CA* and *RICTOR* genes, respectively. We then conducted pathway analysis, which revealed activation of the AKT pathway. Based on this analysis, efficacy of PIK3CA and AKT inhibitors were evaluated in the tumor biopsy-derived cell culture and PDX, and response to the AKT inhibitor AZD5363 was observed both in vitro and in vivo indicating the patient would benefit from targeted therapies directed against the serine/threonine kinase AKT. In conclusion, our study demonstrates that high throughput signaling pathway analysis will significantly aid in identifying actionable alterations in rare tumors and guide patient stratification into early-phase clinical trials.

## Introduction

The application of precision medicine into clinical practice has substantially impacted the management of cancer over the last decade. Next-generation sequencing (NGS) technologies allow the identification of actionable mutations in tumors, to which targeted therapies can be developed with the potential to improve therapeutic index to specifically target the tumor versus normal tissues in contrast to conventional cytotoxics. Increasingly, NGS of patient tumor samples guides patient stratification into clinical trials, such that only the patients bearing specific molecular alterations will receive the corresponding targeted therapy. However, a major hurdle in matching patients to the correct targeted therapies is the lack of driver mutations in a majority of tumors that are sequenced in the clinic. This highlights the need to bring forward cost-effective complementary approaches that will aid in identifying activated pathways that can be targeted therapeutically.

The TARGET (Tumor chARacterisation to **G**uide **E**xperimental Targeted **T**herapy) protocol aims to stratify patients based on genetic alterations identified in tumor specimens and/or circulating-free DNA. In addition, in vivo drug efficacy studies in patient-derived xenografts (PDX) and pathway analysis are performed for a subset of patients. This approach aims to match patients to the most appropriate and available early-phase clinical trials according to their molecular alterations to maximize the chance of patient benefit from targeted therapies^1^.

Here we describe a case study (TAR007) of a patient with no smoking history and a poorly-differentiated neuroendocrine tumor of unknown primary origin. These are rare tumors, characterized by poor prognosis, and these patients have limited treatment options. Chemotherapy and/or radiotherapy treatment, prior to or after surgical resection of detected tumor masses may be utilized, but there is limited data for the use of targeted therapies to treat this type of cancer^2^. In this study, we conducted an extensive molecular characterization of a freshly resected tumor biopsy to identify constitutively activated and druggable cell survival pathways in this tumor specimen. While whole exome sequencing (WES) did not show any actionable mutations, *PIK3CA* and *RICTOR* gene amplifications were detected by whole genome sequencing (WGS). Complementing this approach, we used a high-throughput platform for analysis of cell signaling pathways and detected hyperactivation of the AKT signaling axis. Treatment of the tumor biopsy-derived cell cultures, or a successfully established PDX model showed response to AKT inhibitors, and little or no effect of PI3K inhibitors. These results highlight that combining NGS, signaling pathway analyses, and preclinical drug efficacy studies can successfully identify activated pathways that can be targeted therapeutically in rare tumors that lack actionable driver mutations. In addition, we identify amplified *PIK3CA* and *RICTOR* as potential biomarkers for patients with neuroendocrine tumors with increased propensity to respond to treatment with AKT inhibitors.

## Results

**Fig. 1:**
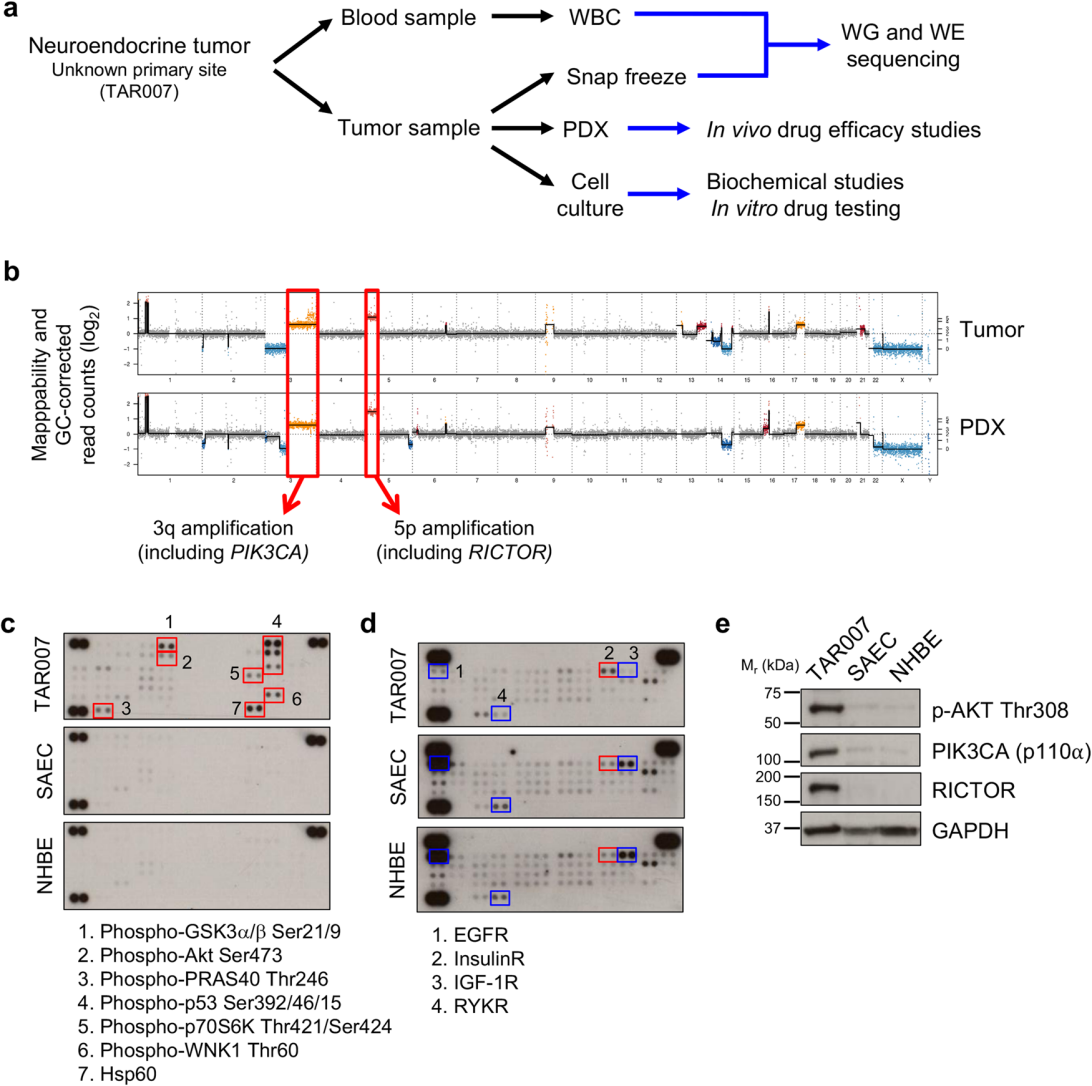
Activation of the AKT pathway in a neuroendocrine tumor of unknown primary origin. **a** Scheme depicting sample collection and processing for the TARGET clinical trial and the studies performed for the molecular analysis of the tumor specimen TAR007. WBC White Blood Cells, WG Whole Genome, WE Whole Exome. **b** Whole genome sequencing from the tumor and a patient-derived sample shows extensive copy number variations, including amplifications in 3q and 5p chromosomes. **c** Phosphokinase arrays from tumor-derived low passage cell cultures show activation of several components of the AKT-mTOR signaling pathway compared to normal primary cells NHBE (normal human bronchial epithelium) and SAEC (small airway epithelial cells). **d** Phospho-receptor tyrosine kinase (RTK) arrays from TAR007 low passage-derived cell cultures show different RTK activation profiles. e Western blot confirming overexpression of PIK3CA (aka p110α) and RICTOR, as well as hyperactivation of AKT in TAR007-derived cell cultures. GAPDH was used as a loading control. Uncropped blots are available in Figure S3

A patient presented with a poorly-differentiated neuroendocrine tumor of unknown primary origin, and the tumor was surgically resected from the right axilla. An experimental pre-clinical study was designed to identify potentially druggable genetic alterations (Fig. 1a). Tumor samples were inoculated into NSG mice to generate a PDX mouse model for use in drug efficacy studies. In parallel, tumor fragments were frozen for DNA sequencing and a low passage cell culture was derived after digestion of the tumor specimen.

WES of the tumor specimen was conducted to identify targetable alterations in this patient’s tumor, and DNA from white blood cells was used as a control to exclude germline variants from the analysis. WES did not reveal any actionable mutations (Supplemental file 1). WGS was then performed to identify copy number alterations. WGS analysis from both the tumor specimen and a PDX-derived sample revealed chromosome deletions at 3p and 14q, and amplifications in 3q, 5p, 13q, and 17q (Fig. 1b). Among the regions of amplification, we detected copy number gains of the *PIK3CA* (in 3q, 4 copies) and *RICTOR* (in 5p, 5 copies) genes, which are upstream activators of the oncogenic kinase AKT.

Fresh tumor biopsy-derived low passage cell cultures were then used to assess pathways activated in the tumor sample. Normal, non-transformed, primary cells were used as controls (NHBE and SAEC; Fig. 1c). Several components of the AKT pathway were specifically activated in the tumor-derived cells, including AKT, PRAS40, and GSK3α/β, consistent with this pathway being activated due to amplification of *RICTOR* and *PIK3CA*. High levels of phosphorylation were also found for the p53 tumor suppressor protein, and the WNK1 protein kinase (Fig. 1c). Using phospho-receptor tyrosine kinase (RTK) arrays, hyperactivation of the insulin receptor was detected. Interestingly, we observed downregulation of the epithelial growth factor (EGF), insulin-like growth factor 1 (IGF-1), and receptor-like tyrosine kinase (RYK) receptors (Fig. 1d). Overexpression of PIK3CA, RICTOR, and activation of the AKT pathway were further confirmed by western blot (Fig. 1e). Furthermore, positive pAKT staining was detected in the patient’s biopsy and PDX models by immunohistochemistry, indicating the AKT pathway was activated, however at lower levels than a PTEN-negative prostate cancer specimen that was used as positive control (Figure S1).

**Fig. 2:**
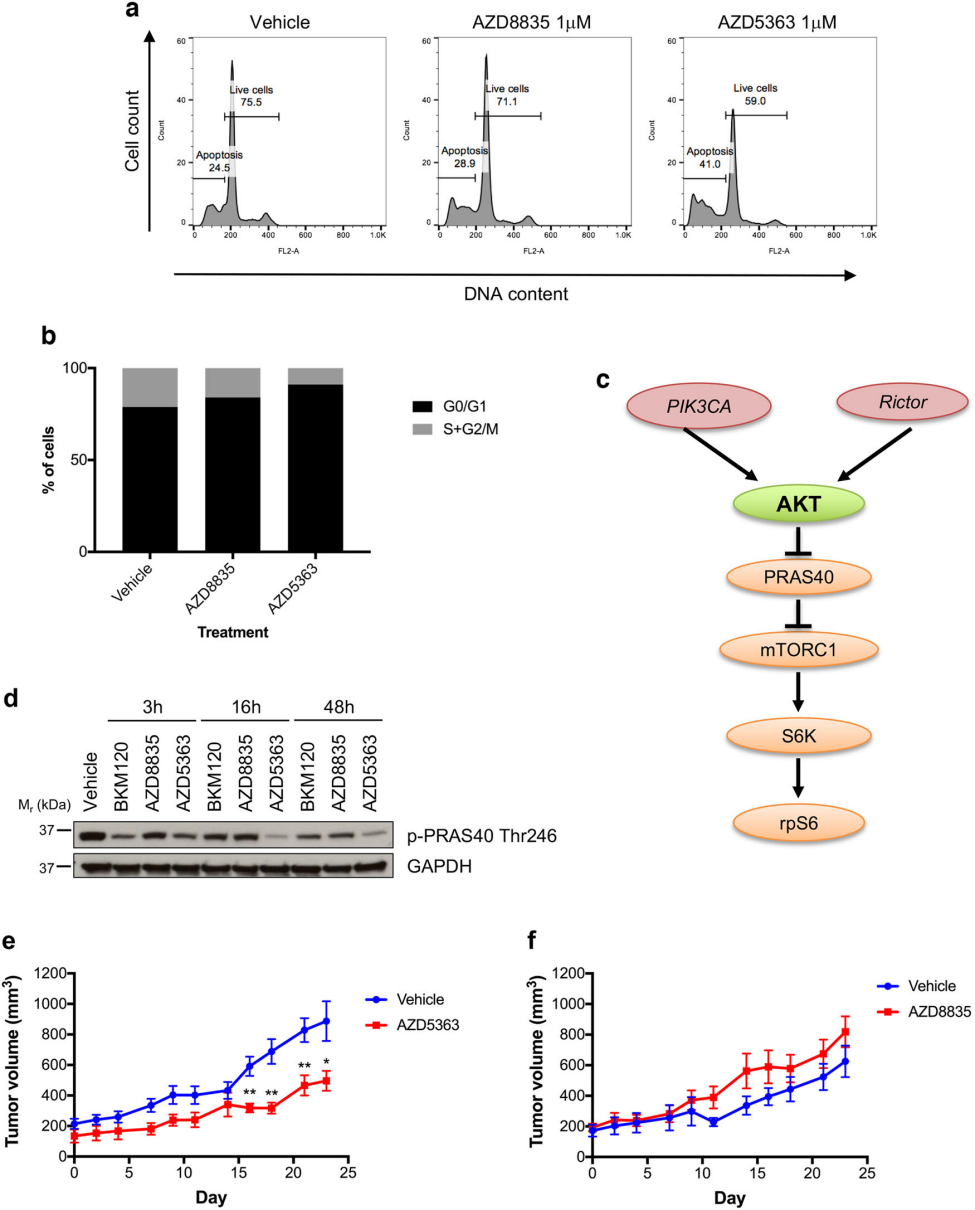
TAR007 patient-derived cancer cells and xenografts (PDX) are sensitive to the AKT inhibitor AZD5363. **a** Tumor-derived low passage cells were treated with the indicated inhibitors for 48 h and DNA content was analyzed by propidium iodide staining and flow cytometry. The percentages of live and dead cells are indicated. Histograms are indicative of 3 independent experiments (*N* = 3). **b** Percentage of cells in each phase of the cell cycle (G0/G1 or S + G2/M) of cells analyzed in **a** (*N* *=* 3 independent experiments). **c** Scheme of the AKT pathway. **d** TAR007 cells were treated at 1 μM concentration with the indicated inhibitors for different times and effectors of the AKT-mTOR pathway were analyzed by western blot. GAPDH was used as a loading control*;* a representative western blot of *N* = 3 independent experiments is shown. Uncropped blots are available in Figure S3. **e**, **f** TAR007 PDX *(N* *=* 9 mice/group) were treated with **e** vehicle and AZD5363 or **f** vehicle and AZD8835 as described in the methods section, for the days indicated and tumor volume was recorded every 48 h. Data are shown as mean ± SEM. **P-value < 0.01; *P-value < 0.05

Our data indicated that the PI3K/AKT pathway was constitutively activated so inhibitors targeting these kinases were explored in functional assays. Treatment of the tumor-derived cells with AZD8835 or GDC0941, two PIK3CA selective inhibitors, had minor pro-apoptotic effects compared to vehicle-treated cells (Fig. 2a and S2A). In contrast, treatment of the tumor-derived cells with two selective AKT inhibitors, AZD5363 (an ATP-competitive inhibitor), or MK2206 (an allosteric AKT inhibitor) resulted in a significant increase in cell death (Fig. 2a and S2A). In addition, AKT inhibition had a strong effect on cell proliferation, and diminished the percent of proliferating cells (21.1% in vehicle-treated cells; 16.0% in AZD8835-treated cells and 9.0% in AZD5363-treated cells; Fig. 2b). Time course experiments were then conducted to investigate mechanisms of sensitivity to AKT inhibitors. Treatment with the AKT inhibitors, AZD5363 or MK2206, resulted in sustained inactivation of the AKT substrate PRAS40, this was not the case with PI3K inhibitors (BKM120, GDC0941, and AZD8835; Fig. 2c, d and S2B). PRAS40 is a negative regulator of mTORC1, a multiprotein complex required for cell growth and proliferation. Phosphorylation of PRAS40 by both AKT and mTORC1 promotes the dissociation of PRAS40 from mTOR, leading to activation of mTORC1 (Fig. 2c)^3^. Decreased phosphorylation of the mTORC1 downstream substrate ribosomal protein S6 (rpS6) was also observed in cells treated with AKT inhibitors, consistent with non-phosphorylated PRAS40 binding and inhibiting mTORC1 activation (Figure S2B). Cells treated with PI3K inhibitors did not display sustained decreased rpS6 phosphorylation (Figure S2B). Therefore, sustained inactivation of pathways downstream of AKT in cells treated with AKT inhibitors is likely to account for the sensitivity observed towards the AKT inhibitors and not to PI3K inhibitors.

We then expanded these in vitro studies to explore responses in PDX models. PDX models resembled the original biopsy histology, as assessed by immunohistochemistry for the neuroendocrine marker CD56 and the proliferation marker KI67. pAKT staining was observed at varying levels in different PDX samples, indicative of intra-tumor heterogeneity (Figure S1A). Consistent with data derived from the low passage cell cultures, treatment with AZD5363 in vivo significantly reduced tumor growth by approximately 50% (Fig. 2e). PI3K inhibition with AZD8835 showed no significant difference in the tumor growth rate when compared with the vehicle-treated group (Fig. 2f). Together our data confirm inhibition of AKT as a viable therapeutic strategy and demonstrate that molecular pathway analysis and PDX mouse models can be used in combination to significantly aid in stratifying patients for treatment with targeted therapies in the absence of known driver mutations.

## Discussion

Here, we report extensive molecular characterization from a high grade neuroendocrine tumor of unknown primary origin with the aim of identifying actionable alterations. Increasingly, NGS approaches, such as WES, are being used in the clinical setting to facilitate identification of actionable mutations. However, in this patient case study, WES analysis of the surgical specimen did not reveal the presence of any targetable genetic alterations. Amplification of chromosomes 3q and 5p were identified by WGS; this included copy number gains in the *RICTOR* and *PIK3CA* genes, which correlated with AKT pathway activation. High throughput technologies analyzing cellular signaling networks, including phospho-arrays, are being implemented into therapeutic target and biomarker discovery programs^4,5^. These approaches aid in identifying signaling networks activated in tumor specimens, as genetic mutations do not always correlate with changes in protein activity. A cost-effective phospho-array analysis was used and revealed hyperactivation of the PI3K/AKT pathway, consistent with amplification of the *RICTOR* and *PIK3CA* genes.

A strength of the TARGET protocol is the ability to simultaneously generate patient-derived preclinical models in which to test the efficacy of potential personalized treatments. PDX mouse models are to date considered to accurately predict tumor responses, however, it can take a prolonged time to expand the tumor specimen in animals before any pharmacological study can be conducted. To overcome this issue, we also established low passage tumor-derived cell cultures to test the efficacy of PI3K and AKT inhibitors based on identification of the activated AKT pathway by the phospho-array and confirmation by Western blot analysis. Increased cell death was observed after treatment with the AKT inhibitors AZD5363 and MK2206, however, little or no response was observed to PI3K inhibitors. This is in agreement with recent data, showing that tumors with *RICTOR* amplification are frequently refractory to PI3K inhibition^6^. Sensitivity to AKT inhibitors might rely on sustained inhibition downstream of AKT and mTORC1, an effect not observed after PI3K inhibition. Patients with *RICTOR* amplification might also benefit from mTOR inhibitors^6^; however, these compounds were not tested since secondary resistance normally appears and involves reactivation of the PI3K-AKT or the MAPK pathways^7,8^. Similar responses to AKT and PI3K inhibitors were observed in our PDX models, indicating that low passage cell cultures, when possible to generate, can be a powerful tool for initial inhibitor testing and aid in refining in vivo experiments. In addition, our data suggest that *PIK3CA* and *RICTOR* gene co-amplification may define a unique subset of patients with neuroendocrine tumors that will respond to AKT inhibitors, and could thus be used as an additional biomarker for treatment stratification.

In this particular patient case there were no available early-phase clinical trials with an AKT inhibitor recruiting patients with neuroendocrine tumors. A lack of available relevant clinical trials to cover all possible identified alterations has been recognized as a challenge in many precision medicine programs and is a key reason for only ~5–15% of patients with detected actionable alterations being matched to relevant targeted therapies^9^; https://dctd.cancer.gov/majorinitiatives/NCI-sponsored_trials_in_precision_medicine.htm. The technological ability to identify actionable targets has outpaced the speed of change in clinical trial design where a more flexible structure is needed to facilitate successful delivery of precision medicine. We are exploring a framework to this effect within our own institution for such unique cases where comprehensive patient-derived pre-clinical data is available and where no suitable clinical trial or agent is otherwise available.

To date enrollment into clinical trials mostly relies on mutational status of the corresponding target (e.g., AKT). Our study suggests that complementation of sequencing approaches with cost-effective and time-efficient antibody-based technologies, such as phospho-arrays, can maximize the detection of druggable pathways in tumor cases where no actionable mutations have been identified or little is known about tumor etiology. Lack of detection of actionable mutations by sole use of genomic techniques is a frequent problem in the clinic; recent precision medicine trials, including the IMPACT and MOSCATO trials, have reported 44% and 49% of patients with actionable mutations respectively^10,11^. In addition, a recent cancer genomics study has shown that half of driver mutations in tumors occur outside of well-characterized cancer genes^12^; such mutations will not be identified in tumor sequencing studies. Therefore, clinical implementation of these unbiased signaling pathway analysis technologies to large patient cohorts or rare tumor cases, such as the one presented in this study, could improve selection of patients to early-phase clinical trials of targeted therapies. Such need to broaden the spectrum of techniques in precision oncology to detect genetic and non-genetic targetable cancer vulnerabilities has been recently raised^13^. Integration of these technologies into the clinic coupled with modern trial designs that can adapt to the ‘N of 1’ scenario will aid precision medicine-based approaches.

## Methods

### Patient details

A 68-year old gentleman presented with an isolated right axillary mass in October 2014. An axillary biopsy revealed poorly-differentiated (Grade 3, Ki67 85%) neuroendocrine carcinoma of unknown origin (positive for CD56, chromagranin and synaptophysin; negative for TTF1, CDX2 and Merkel Cell Polyomavirus). Positron emission technology/computed tomography demonstrated a right axillary mass and no other identifiable sites of disease. The patient received six cycles of carboplatin and etoposide chemotherapy between Oct 2014 and April 2015 with RECIST partial response after three cycles but with evidence of tumor growth after cycle 6. He was referred to the Experimental Cancer Medicine Team and consented to TARGET in June 2015. In parallel he was referred for surgical resection of the isolated axillary mass which achieved a complete resection and permitted access to fresh tissue for PDX and translational research in July 2015.

Following a brief disease-free period, the patient relapsed with metastatic nodules within the retroperitoneum in December 2015. The patient was treated with capecitabine and temozolamide between March and October 2016 with best response of progressive disease. Treatment was switched to interferon and sandostatin and his disease has remained stable by RECIST 1.1 criteria on this combination at the time of writing.

### Ethical approval

The TARGET protocol and associated translational research analyses were approved by the North-West Preston Research Ethics Committee, United Kingdom in February 2015. The patient provided fully informed written consent to participate in this trial and for the use of donated tumor/blood samples for research that may help identify personalized therapeutic options. A blank consent form is provided in the supplementary information section. Research conducted in this study was performed in accordance with the Human Tissue Act 2004, and regulations approved by the Human Tissue Authority at The Christie NHS Foundation Trust (Manchester, UK). All patient information has been anonymized.

### DNA isolation

DNA was extracted from snap frozen tumor specimen, patient-derived xenograft fragment and tumor-derived cells in culture using the AllPrep DNA/RNA Mini Kit (Qiagen) according to the manufacturer’s protocol.

### Whole exome sequencing

Genomic DNA (1 μg) was sheared in a Covaris S2 ultrasonicator (Covaris Inc) to an average size of 150–200 bp. Multiplexed libraries were prepared using the SureSelectXT Target Enrichment System for Illumina Paired-End Sequencing Library kit and the SureSelect Exome V6 + COSMIC Capture Library (Agilent). Library quality was checked using the Agilent Bioanalyzer. Libraries were quantified by qPCR using the KAPA Library Quantification Kit for Illumina (Kapa Biosystems Inc.). 1.8 pM pooled libraries were loaded onto the NextSeq 500 and 2 × 76 bp sequencing was carried out using a NextSeq 500/550 High Output v1 kit (Illumina Inc.).

### Whole genome sequencing

Genomic DNA (300 ng) was sheared in a Covaris S2 ultrasonicator (Covaris Inc) to an average size of 150–200 bp. Multiplexed libraries were prepared using the NEBNext DNA Ultra kit (New England Biolabs) with an input of 50 ng sheared DNA. Library quality was checked using the Agilent Bioanalyzer. Libraries were quantified by qPCR using the KAPA Library Quantification Kit for Illumina (Kapa Biosystems Inc.). 1.8 pM pooled libraries were loaded onto the NextSeq 500 and 2 × 151 bp sequencing was carried out using a NextSeq 500/550 Mid Output v2 kit (Illumina Inc.).

### Sequencing data processing

Reads were quality checked and aligned to GRCh37/hg19 reference genome using BWA (v0.7.7)^14^ with default options. Prior to downstream analysis, samtools (v1.2)^15^ were used to retain only uniquely mapped reads. BAM files were coordinately sorted and de-duplicated via Picard (v1.96).

### Calling variants

Variants were called using GATK (v.3.1.1)^16^ based upon established best practices^17^. Prior to calling variants, local realignment around known indels was performed on the dedupped, sorted BAM files using realignment targets from known sites (e.g., dbSNP, 1000 Genomes). Realignment around indels helps improve the accuracy of the downstream processing steps. In order to detect systematic errors made by the sequencer when it estimates the quality score of each base call, base quality score recalibration was performed. These steps generate the BAM files ready for variant calling. Variants were called using the UnifiedGenotyper on the target regions only. The call set produced by GATK was filtered for required variants using custom scripts. For variant annotation, ANNOVAR^18^ was used.

### Copy number analysis

HMMcopy (v0.99.0)^19^ was applied to the dedupped BAM files to detect copy number change. Briefly, the genome was divided into windows of fixed size (150 kb) and read count was determined as the number of reads overlapping each window. GC content and mappability bias correction were performed on tumor and normal samples, filtering out GC-content within the top or bottom 1% quantile. The remaining windows with mappability score greater than 0.9 were kept. The corrected read counts in each bin were used to calculate the log_2_ratio. A 6-state Hidden Markov model (HMM-based) approach was used to segment the data into regions of similar copy number profile and to predict a copy number alteration event (i.e., 0, 1, 2, 3, 4 or > 5 copies of chromosome) for each segment.

### Animal research

Animal research was approved by the Cancer Research UK-Manchester Institute Ethical Review Committee, and was performed in accordance with the Animals (Scientific Procedures) Act 1986 and ARRIVE (Animal Research: Reporting of In Vivo Experiments) approved guidelines and regulations. PDX were generated as previously described^20,21^. Six to eight week-old Nod.*scid*.IL2γ (NSG) mice were obtained from Charles River. Tumor volumes were calculated according to the formula: tumor volume = (length × width^2^)/2. When tumors reached 150 mm^3^, mice (*N* = 9 per group) were randomized and drug/vehicle treatments were started. AZD5363 and AZD8835 were administered by oral gavage at a dose of 150 and 75 mg/Kg, respectively, as previously described^22,23^. Mice were culled when tumor volumes reached 800–1000 mm^3^ or after 6 months if there were no signs of tumor growth.

### Low passage cell cultures

A tumor fragment (~1 cm^3^), from the patient’s original biopsy, was minced and digested with type I collagenase (100 U/mL; 16 h) in serum-free, 2 × Penicillin/Streptomycin RPMI1640; and washed with cold ACK lysis buffer (Gibco). Cells were filtered through a 40 μm cell strainer and seeded in HITES medium supplemented with 1% FBS (Labtech). After 24 h, two populations were distinguished: an adherent one with fibroblast morphology which was frozen, and a suspension one which was used for further studies and corresponded with tumor material. Cultures were maintained in HITES for a maximum of 4 weeks (3–4 passages). HITES medium was prepared by mixing RPMI1640 with Insulin-transferrin-selenium-ethanolamine and hydrocortisone (10 nM, Sigma). All solutions were purchased from Gibco unless indicated. SAEC (small airway epithelial cells) and NHBE (normal human bronchial epithelial) cells were purchased from Lonza in February 2016 and maintained as recommended for two passages.

### Phospho-arrays

The Proteome Profiler Human Phospho-Kinase and human phospho-receptor tyrosine kinase (RTK) Array kits (R&D Systems) were used according to the manufacturer’s instructions.

### Reagents and drug treatments

AZD8835 and AZD5363 were kindly provided by AstraZeneca. BKM120, GDC0941, and MK2206 were purchased from Selleckchem. Drugs were resuspended in DMSO (10 mM stocks) and used at 1 μM for the times indicated.

### Cell cycle analysis

Cells were seeded (2 × 10^5^ cell/mL) and treated with the corresponding inhibitors for 48 h. Cells were then collected and treated with trypsin (Gibco) for 5 min to disperse cell clumps, and spun at 2000 rpm for 3 min. BD Cycletest Plus DNA Reagent Kit was used for sample staining, according to the manufacturer’s protocol. Samples were run on a BD LSR Fortessa, and results analyzed using FlowJo.

### Western blot

For western blot, cells were lysed as previously described^24^. Antibodies to phospho-AKT Thr308 (#2965), phospho-PRAS40 Thr246 (#2997), phospho-ribosomal protein S6 (rpS6) Ser240/244 (#5364), p110α/PIK3CA (#4249), and GAPDH (#2118) were from Cell Signaling technologies; Rictor (A300-459A) was purchased from Bethyl Laboratories and Tubulin (T9026) was from Sigma. All samples loaded in a corresponding gel derive from the same experiment and were processed in parallel. Uncropped blots are available in Figure S3.

### Immunohistochemistry

Formalin-fixed, paraffin-embedded sections from the patient biopsy and PDX were stained by immunohistochemistry (IHC) for CD56 (NCL-CD56-aB6, Novocastra), KI67 (Dako) and p-AKT Ser473 (Cell Signaling technologies #3787). Citrate antigen retrieval was used. Incubation with primary antibodies, according to manufacturer’s protocol, was followed by incubation with HRP-coupled secondary antibodies and development with diaminobenzidine and hematoxylin counterstaining following standard procedures^25^.

### Statistical analysis

Statistical analysis was done with GraphPad Prism, the Mann–Whitney test was used to assess differences between treatments; when *P*-value < 0.05, differences were considered significant.

### Data availability statement

All data generated or analyzed during this study are included in this published article and its supplementary information files.

## Electronic supplementary material

Supplemental material

Supplemental File 1
