# Supplementary material for: Signaling pathway screening platforms are an efficient approach to identify therapeutic targets in cancers that lack known driver mutations: a case report for a cancer of unknown primary origin
Source: NPJ Genom Med. 2018 Jun 20;3:15. doi: 10.1038/s41525-018-0055-6 (PMC6010465; doi:10.1038/s41525-018-0055-6)
Supplement: Supplementary file 1 — Supplemental material [file 41525_2018_55_MOESM1_ESM.pdf]

SUPPLEMENTARY INFORMATION

Supplemental figures

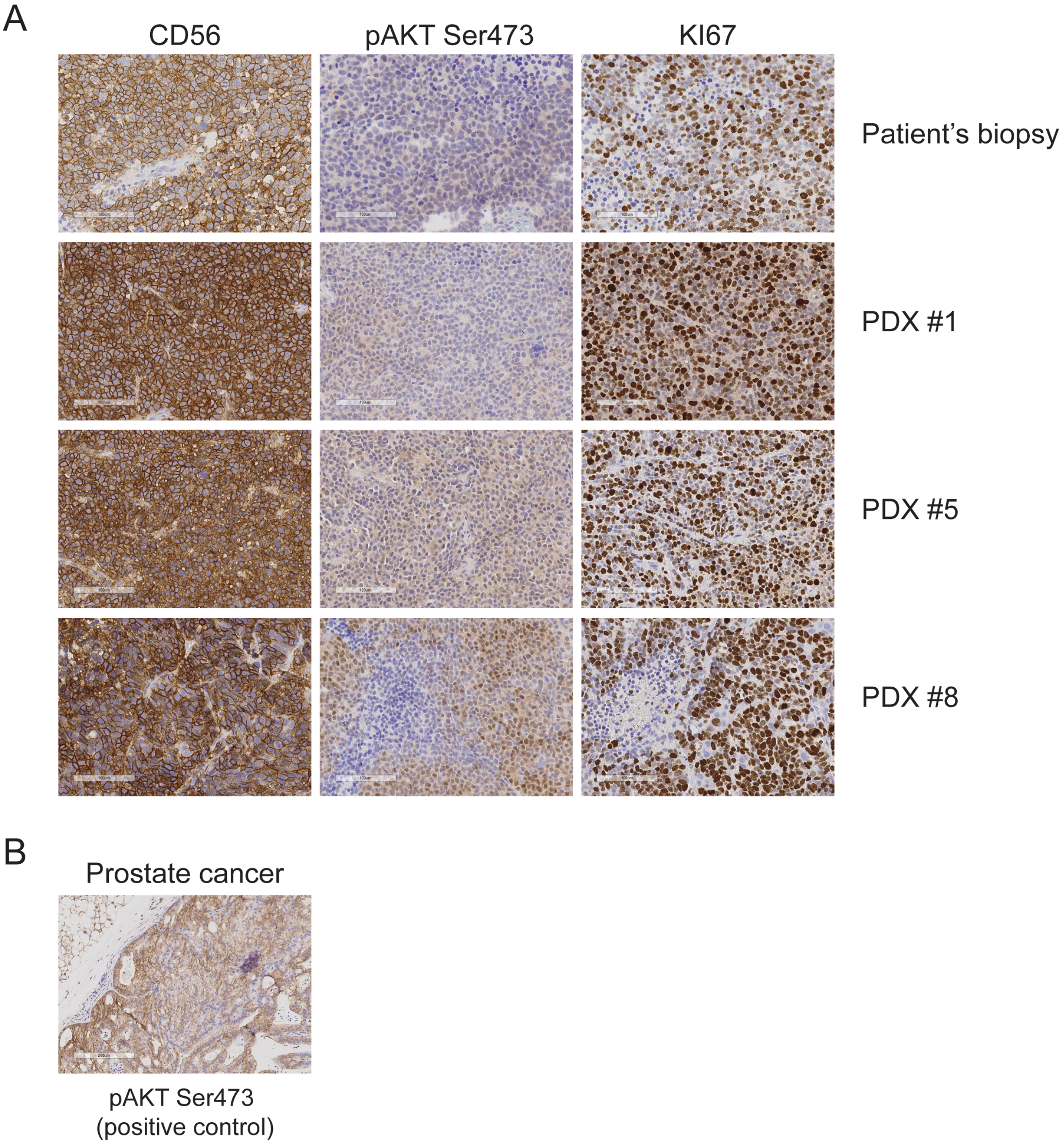

Figure S1. (A) Formalin-fixed paraffin-embedded samples of matched TAR007 biopsy and matched patient derived xenograft (PDX) models were stained with the corresponding antibodies. Incubation with primary antibodies, was followed by incubation with HRP-coupled secondary antibodies and development with diaminobenzidine and hematoxylin counterstaining. (B) A PTEN-negative prostate cancer sample was used as

pAKT Ser473 staining positive control. Images were acquired at 20x magnification (bar = 100μm).

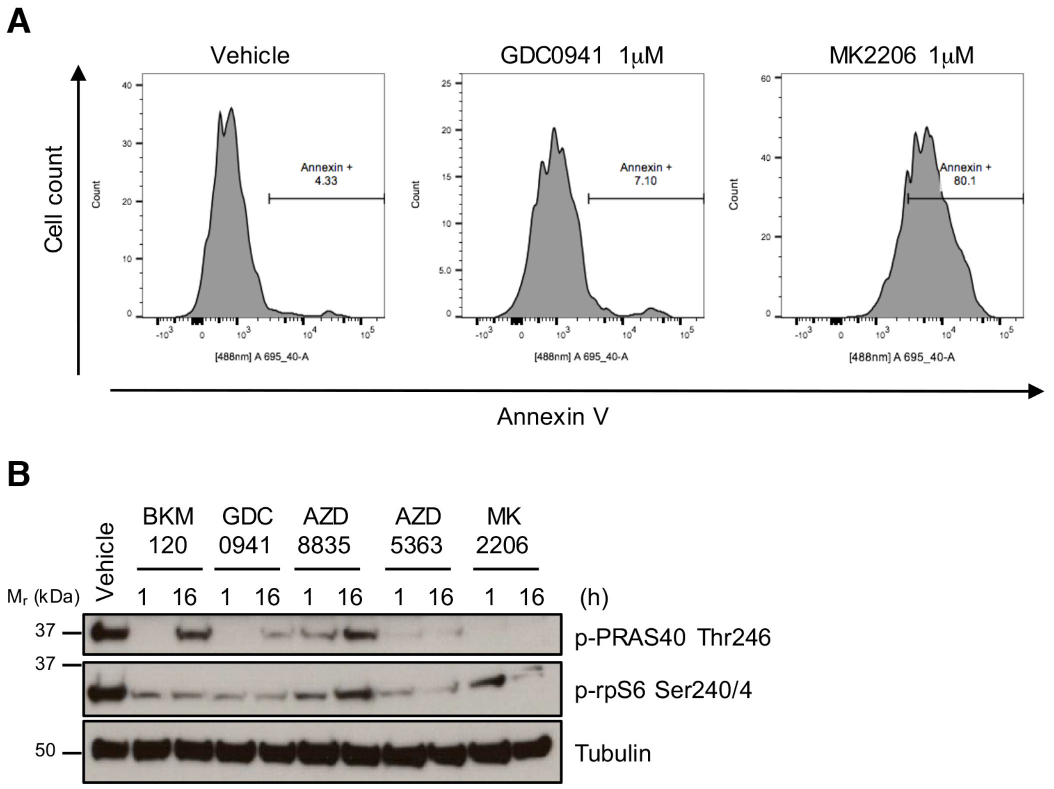

Figure S2. (A) Tumor-derived low passage cells were treated with the indicated inhibitors for 48h and apoptosis was analyzed by Annexin V-APC staining (BD biosciences; according to the manufacturer's protocol) and flow cytometry. The percent of Annexin V positive (apoptotic) cells is indicated. *N* = 2 experiments. (B) TAR007 cells were treated at 1μM concentration with the indicated inhibitors for different times and effectors of the AKT-mTOR pathway were analyzed by western blot. Tubulin was used as a loading control; *N* = 2 experiments.

Corresponding to Fig. 1C

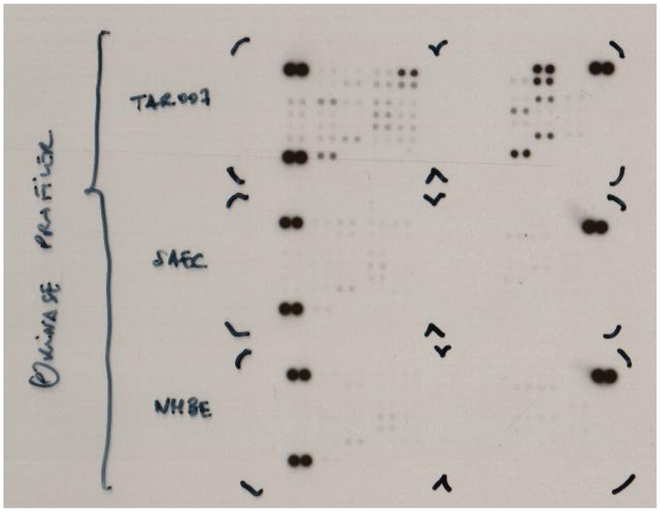

Corresponding to Fig. 1D

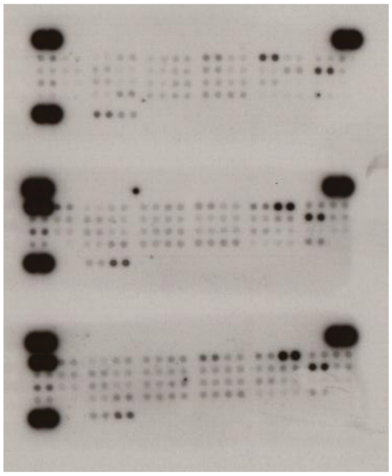

TAR007

SAEC

NHBE

Corresponding to Fig. 1E

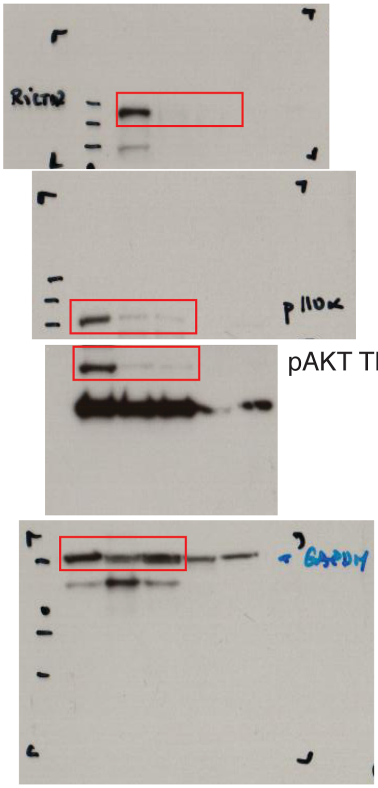

Corresponding to Fig. 2D

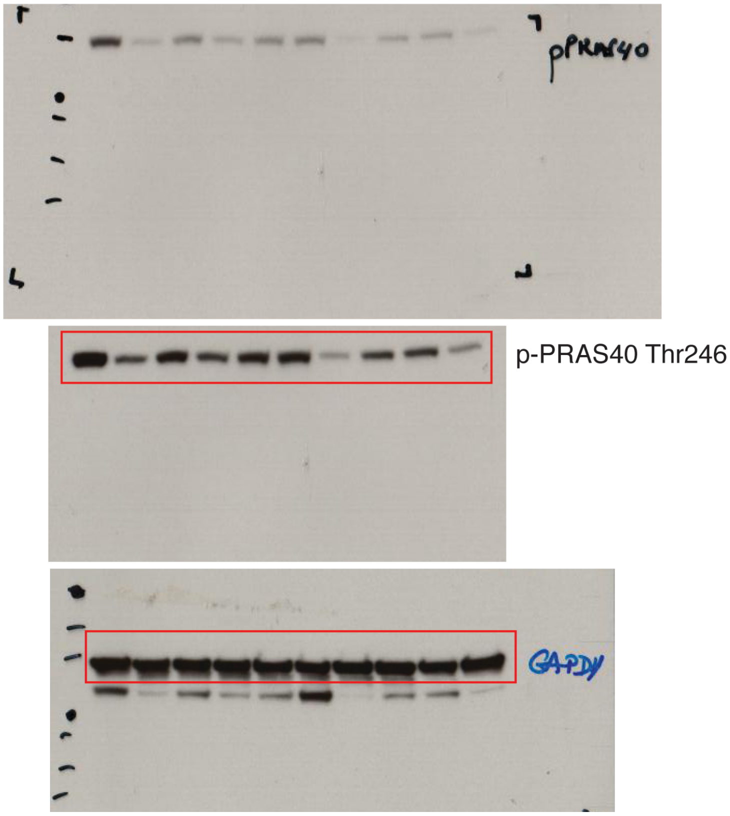

Figure S3-1. Each membrane was cut into several parts according to molecular weight, and each part was incubated with specific antibody as indicated.

Corresponding to Fig. S2B

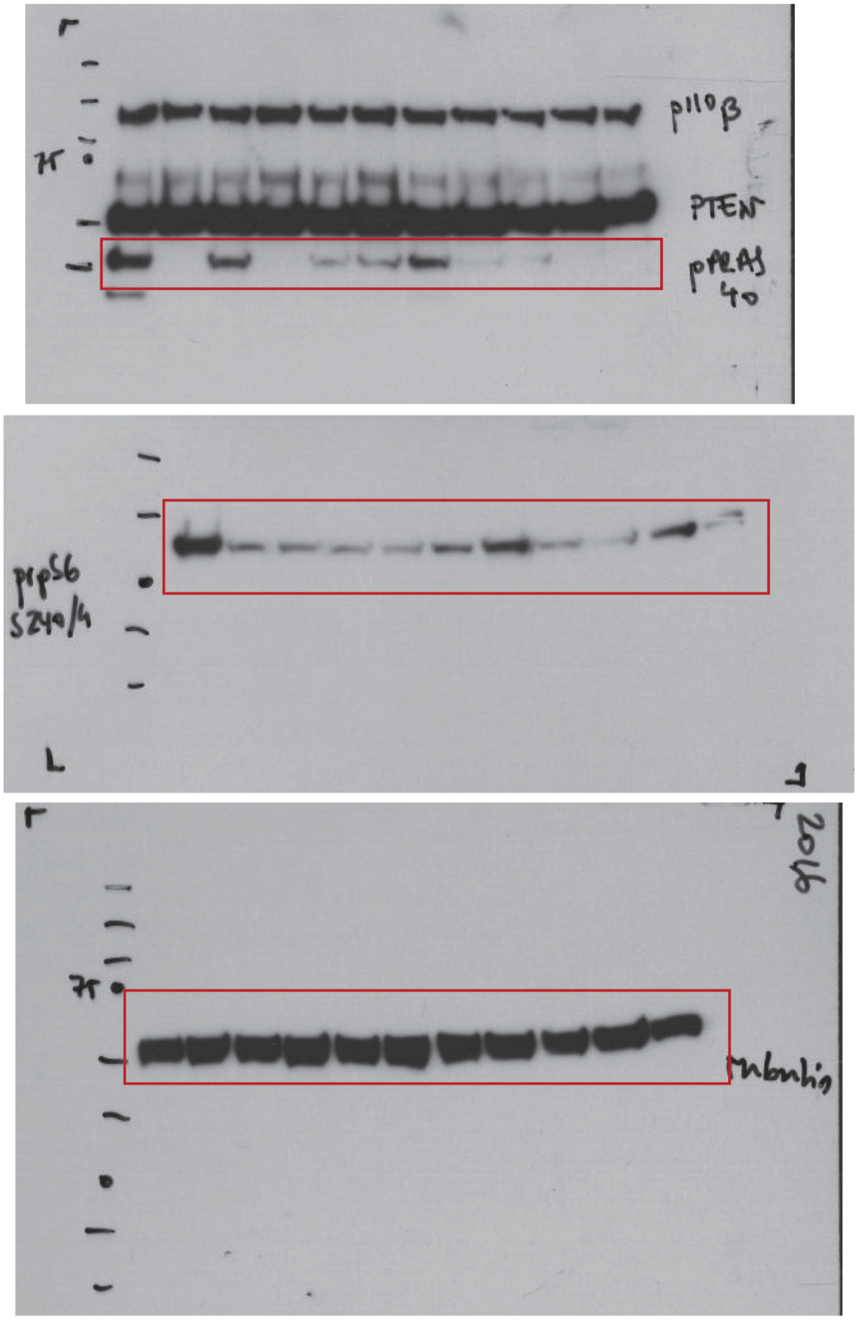

Figure S3-2. Each membrane was cut into several parts according to molecular weight, and each part was incubated with specific antibody as indicated.

Ethical approval

A blank consent form, identical to the one approved by the patient, is provided at the end of the document.

Supplemental File 1

Excel file containing Whole Exome Sequencing results from TAR007 sample.

The Christie NHS
NHS Foundation Trust

We Care, We Discover, We Teach

PARTICIPANT INFORMATION SHEET AND CONSENT FORM

Manchester Cancer Research Centre
TARGET

Title: TUMOUR CHARACTERISATION TO GUIDE EXPERIMENTAL TARGETED THERAPY - THE 'TARGET' STUDY

You are being invited to take part in a research study. Before you decide it is important for you to understand why the research is being done and what it will involve. Please take time to read this information carefully. Talk to others about the study if you wish.

Part 1 tells you about the purpose of the study and what will happen if you take part.

Part 2 gives you more detailed information about the conduct of the study

Please ask us if there is anything that is not clear to you or if you would like more information. Take time to decide whether or not you wish to take part.

PART.1.

What is the purpose of this study?

The purpose of this study is to analyse the genetic characteristics of your cancer and to provide results to you and your doctor that may help inform your suitability to receive an experimental treatment. Each cell in your body contains a copy of your genetic code, made up of DNA. In cancer cells the DNA is often faulty and this allows the cells to grow out of control. Understanding these genetic faults, may help your doctor to select a specific experimental treatment for you within a clinical trial that aims to treat the fault. More and more, cancer treatments are moving away from chemotherapy to newer treatments that 'target' specific faults within cancer cells and which potentially cause fewer side effects on non-cancer cells. If an appropriate treatment trial is available for you, you will be given further details and asked to sign a separate consent form to take part in that trial.

We can analyse the genetic code of your cancer by analysing the small amounts of DNA which the tumour leaks into the blood stream or from cancer cells circulating in the blood. This is a primary goal of the study to use blood samples to analyse the genetic code of the tumour and so in the future avoid the need for patients to undergo a biopsy of their tumour. To understand whether the blood sample is representative of the tumour we would like to compare this using either a sample of tumour tissue taken from a previous biopsy or, if you are agreeable and if it is safe to do so, by performing a new biopsy.

We cannot guarantee that we will find a fault in the genetic code of your cancer, or that if we do there will be a suitable drug trial for you. However, as we learn more about the genetics of cancer in this study, it will help doctors and scientists develop new treatments to help people in the future.

We will also ask your permission to grow your tumour samples or cancer cells from the blood in the laboratory. This may involve work with animals. This will help doctors and scientists learn more about how cancers grow and help to develop new treatments for cancer patients in the future. You can opt out of this part of the study if you prefer.

Why have I been invited?

You have been invited because you have a diagnosis of cancer and you are being considered for experimental drug treatments. In order to help your doctor select a suitable clinical trial for you it is useful to look at the genetic code of the cancer.

Do I have to take part?

Taking part in this study is completely voluntary. We will describe the study and go through this information sheet, which we will give to you. If you are agreeable, we will then ask you

to sign a consent form to show you have agreed to take part. You are free to withdraw at any time, without giving a reason. This would not affect the standard of care you receive.

What will happen to me if I take part?

A member of the clinical study team will explain the study to you and determine if you are eligible to take part. You will be provided with this information leaflet about the study and will have ample time to read the information and discuss it with your family or friends if you wish. You will then need to complete the informed consent form in order to participate.

We will ask your permission to provide the following samples, some of which are a requirement for the study and some of which are optional.

Required:

1) You will be asked to donate a sample of your archival tumour tissue, taken from a previous biopsy or operation.
2) You will be asked to provide blood samples (up to 60 ml, roughly 4 tablespoons) at the beginning of the study. Every effort will be made to take the blood samples at the same time as your routine blood tests at clinic visits. The extra blood samples will not increase the amount of time you spend in hospital.
3) If you are subsequently enrolled onto a clinical study to receive an experimental medicine designed to 'target' specific genetic faults identified in your tumour you will be asked for additional blood samples on up to 5 occasions (up to 60 ml, roughly 4 tablespoons, each time) during your treatment. Every effort will be made to take the blood samples at the same time as your routine blood tests at clinic visits.
4) If you need to undergo an operation or biopsy in the future (as part of your routine care) we will ask your permission to obtain any leftover samples that are surplus to diagnostic requirements. This may also include samples such as fluid removed from around the lung or abdomen, or possibly a bone marrow biopsy, if applicable to you.
5) You will be asked to consent for information to be collected about you such as your date of birth, diagnosis and previous treatments. If you are subsequently enrolled onto a clinical study to receive an experimental medicine we will collect information about how well you benefit from that treatment and for how long. This information will be obtained by telephone contact with you or your GP or from your health records. We will monitor for up to 5 years.
6) You will be asked to consent to storage of the tissue and blood samples for research now and in the future for studies aiming to improve knowledge and treatment of cancers.
7) You will be asked for permission for us to examine the genetic code in your normal healthy cells (from a blood sample described above) in addition to that of the cancer. This is important to help us work out which are the faulty genes in the cancer compared with your normal genetic code.

Optional

1) We may ask your permission to obtain a sample of your cancer by a fresh biopsy. You will only be asked to undergo a biopsy if your study doctor determines this can be done with minimal risk and discomfort to you. A biopsy usually involves inserting a small needle into an area of cancer (under local anaesthetic) or a minor surgical procedure. Your study doctor will explain the details of the procedure to you, depending on how the biopsy will be obtained and the person performing the biopsy will ask you to sign a separate consent form. We will perform this biopsy at the beginning of the study.
2) If you are subsequently enrolled onto a clinical study to receive an experimental medicine we will ask your permission to obtain up to 2 further fresh biopsies (if safe to do so). One of these would be performed during treatment and one at the end, if the new treatment stops working. If you need to have a biopsy performed for the clinical trial of the experimental medicine, we would do this at the same time so you are not subjected to repeated biopsies.
3) You may be asked to donate a sample of your urine or hair follicles for analysis.
4) You will be asked if we can use your tumour and/or blood samples to grow cancer cells in the laboratory which may involve experiments with animals (mice).

What tests will be performed on my samples and where will this happen?

We will analyse genetic material (DNA/RNA) from your tissue and blood samples. Specifically the study includes a direct comparison of the genetic code within the tumour cells and your normal cells. The reason for this is to determine what has changed in the cancer and whether this might influence your doctor's decision for selecting an experimental clinical trial for you. However, we cannot guarantee that we will find any abnormalities or if we do whether this will be relevant in selecting a suitable experimental clinical trial for you.

Your samples will be analysed in laboratories in Manchester. This will include the Cancer Research UK Manchester Institute and The Manchester Centre for Genomic Medicine at St Mary's hospital. All your samples will be coded so that laboratory staff are not aware of your personal details. Only doctors and key study personnel will have access to this information. Sometimes, your samples will be shared with our collaborators both within and outside of the UK (Europe or worldwide) if they have expertise to perform certain tests. If your samples are shared for the purposes of research we will not reveal any of your personal identifiable information. However, if we are asking for a diagnostic test from our collaborators we may need to provide your clinical details as we would for any clinical test.

In addition to analysing your DNA/RNA we may also perform other tests on your samples to help us determine why the cancer has developed.

Will the researchers give me results?

The main reason for performing this research is to find genetic faults in your cancer that may help guide your doctors to select an experimental clinical trial, based on available information and their best clinical judgement. A scientific panel consisting of experts in cancer, genetics and ethics from the University of Manchester and The Christie NHS Foundation Trust will serve on a 'Molecular Tumour Board' that will review the results and determine whether the results are important. We will always tell you about results that have a direct impact on the care of your current cancer. If we find something of uncertain significance we will not disclose this to you as we don't know its meaning.

As part of this research we will also be analysing DNA from your normal cells. We call this the 'germline' DNA. It is possible that we may find other results during this study that are indicative of hereditary conditions. In other words we may incidentally find that you or your family members are at higher risk of developing cancer or other medical conditions. You may or may not wish to know this information. Some people may find this information overwhelming and wish not to know these incidental findings, while others will want to know everything. **Our default will be to give you all information. However, if you would prefer not to receive it, you will need to sign the relevant box on the attached consent form stating that you would not wish to receive this information.**

There are two kinds of information that **do not** have direct impact on your cancer but which you may or may not wish to know about:

i) **Results that may have significance for your biological family members**. For example, we may discover that you have a gene that, if inherited by your biologically-related family members, could increase their risk of cancer or other medical conditions. Your family members may or may not have inherited that gene – they would have to be tested to find out. It is important to consider whether you and/or your family would want to know about this.

ii) **Results that are not related to your cancer, but may have potential medical impact for you**. For example, we may discover a gene that increases your risk of another medical condition, not related to your cancer. We cannot know what that condition might be before the testing. It is important to consider whether you would want to receive this information.

We will discuss these situations and provide you with genetic counselling so you are aware of the potential implications of these results. You may also wish to discuss this with your family. We would offer to refer you or your family members to NHS genetic services for further discussion about the implication of the findings should one of these abnormalities be found and should you choose to receive the result. The genetic specialists may offer genetic screening to you or your family members.

What are the possible benefits of taking part?

With this research we may find genetic faults that have direct impact on the management of your current cancer. If we do not find any information that would be important for your cancer you will not receive any personal benefit from taking part. However, the samples that you donate will be used for research to determine more about the genetics of cancer that may help doctors and scientists identify new treatments for patients with cancer in the future. It's also important to note that for the first 20 patients recruited on this study we **may not** be able to feedback any results. These samples will be very important for us to fully optimise the laboratory test and will help us make sure everything is working properly before we can use the information to make treatment decisions for subsequent patients. If you are one of the first 20 patients you will be allowed to take part in the study again once all the tests are established. We will ask you to sign another consent form to do this.

What are the possible disadvantages and risks of taking part in this study?

Blood samples: Blood will be taken by trained nurses and phlebotomists who are experienced in the procedure. The risks involved in donating blood samples are the same as for routine blood tests. There may be discomfort or pain in the skin and tissue around the vein where the blood is taken. There may be bruising over the vein after the procedure and occasionally people may experience light-headedness or fainting.

Fresh biopsy specimens: Biopsies are routinely performed at The Christie by experienced medically trained personnel using techniques that minimise the risks of the procedure. However, as with any procedure there are risks and discomforts. You may feel some pain or discomfort including slight stinging when local anaesthetic is injected to numb the area, swelling or infection at the site where tissue is removed. Possible methods for obtaining the biopsy include CT-guided biopsy, ultrasound-guided biopsy or bronchoscopy. If you choose to allow collection of fresh tissue, your doctor will explain the exact procedure required and potential risks involved. You will be asked to sign a separate consent form at the time the biopsy is performed following an in-depth discussion of the procedure.

CT-guided biopsies use x-rays to show the correct location for the tissue removal. Exposure to x-rays can increase the risk of another cancer developing later in life. The amount of x-rays used to guide a biopsy procedure is similar to what you would receive during the routine scans you have for monitoring your condition. This level of exposure is considered a low risk.

Risks of genetic research: Some genetic information may cause you psychological or emotional distress, (for instance discovery of previously unknown health problems or findings that you could carry a gene for certain diseases). We therefore offer genetic counselling advice before you take part in the study during the informed consent process.

Hair follicles – there may be some slight discomfort whilst taking hair follicle samples, usually from your scalp.

Expenses and Payments

Travel expenses can be provided to you (upto £40 per visit) if you are required to attend an additional visit to the hospital for this study. This is most likely to happen if you undergo a fresh tumour biopsy; other samples will be collected at the same time as your usual hospital visits. We are otherwise unable to pay you for participating in this study.

Will my taking part in the study be kept confidential?

Yes. We will follow ethical and legal practice and all information about you will be handled in confidence. The details are included in Part 2.

What if there is a problem?

We aim to address all complaints as soon as possible if you are not happy with the way you have been treated or if you feel you have suffered any harm during this study. Detailed information on this is given in Part 2.

This completes Part 1 of the information sheet.

If the information in Part.1 has interested you and you are considering participation, please read the additional information in Part 2 before making any decision.

Part.2.

What if relevant new information becomes available?
Sometimes during the course of a research project, new information becomes available. If this information is relevant in any way to you or the management of your cancer we will let you know.

What will happen if I don't want to carry on with the study?
Participation in this study is entirely voluntary. You can withdraw from the study at any time. If you withdraw from the study, you can allow us to use samples collected up to the time of withdrawal and to continue to obtain information from your health records. Or, you can request that we destroy all your identifiable samples and/or that we do not collect any further information from your health records. Some of your samples may already have been processed and analysed and it would not be possible to remove this information so completed test results will be retained.

What if there is a problem?
If you have a concern about any aspect of this study, you should ask to speak to the researchers who will do their best to answer your questions. Contact details are provided at the end of this document. If you remain unhappy and wish to complain formally, you can do this through the NHS Complaints Procedure. Details can be obtained from The Christie hospital Patient Advice and Liaison Service (PALS).

In the event that something goes wrong and you are harmed during the research study there are no special compensation arrangements. If you are harmed and this is due to someone's negligence then you may have grounds for legal action for compensation against The Christie NHS Foundation Trust but you may have to pay your legal costs. The Christie NHS Foundation Trust is covered by NHS Indemnity.

Will my taking part in this study be kept confidential?
Yes. All information that is collected about you during the course of the research will be kept strictly confidential. Procedures for handling your data will be compliant with the Data Protection Act 1998. Research data is kept separate from your main health record and is stored in a locked office and in a password-protected database.

Your samples will be handled and stored in a manner in which your personal identification is not known to laboratory staff. Specimens will be labelled with a research study code.

Data collected during the study may be transferred for the purpose of research studies and analysis to associated researchers within/outside the European Union. Some countries

outside Europe may not have laws that protect your privacy to the same extent as the Data Protection Act in the UK or European Law. Therefore, your name and address will be removed from data before transfer and nothing that could reveal your identity will be disclosed to collaborators conducting research studies or analysing data in academic or commercial organizations elsewhere in the UK or outside the UK.

Involvement of your General Practitioner (GP)

We will inform your GP of your participation in this study and any results that may be directly relevant to the management of your cancer. If you choose to receive information about other findings that may be relevant to you or your family members we will also inform your/ your family member's GP as he/she may need to be involved in addressing any implications of this. If you choose not to receive the additional information then we will not inform your GP of these results either.

What will happen to any samples I give?

Samples will be stored in a secure, limited access area of the Cancer Research UK Manchester Institute within the MCRC Biobank.

Procedures for handling, processing, storage and destruction of samples will be compliant with Good Clinical Practice for Laboratories and the Human Tissue Act 2004. All data will be handled according to the Data Protection Act 1998.

Your samples will be analysed in laboratories in Manchester. This will include the Cancer Research UK Manchester Institute and The Manchester Centre for Genomic Medicine at St Mary's hospital. Sometimes, your samples will be shared with our collaborators both within and outside of the UK (Europe or worldwide) if they have expertise in certain tests. If your samples are shared for the purposes of this research we will not reveal any of your personal identifiable information.

If you give your consent, any unused samples at the end of this study will be gifted to the Manchester Cancer Research Centre (MCRC) Biobank for use in future research projects. The MCRC Biobank make samples available to all kinds of researchers, including pharmaceutical companies for all kinds of cancer research, including genetic studies. Access to Biobank samples is strictly regulated and your samples will only ever be released for high quality cancer research projects approved by the MCRC Biobank Access Committee. Researchers using samples will only ever receive anonymised data and your personal identifiable information will kept strictly confidential.

What will happen to the results of the research study?
Individual results will be provided to you as detailed in Part 1 of this information sheet. The broader scientific results of the study will be published in medical journals and presented at national/international oncology conferences. You will not be identified in any report or publication.

Who is organising and funding the research?
This study has been organised and funded by the Manchester Cancer Research Centre (MCRC). The MCRC encompasses a partnership between The University of Manchester, The Christie NHS Foundation Trust and Cancer Research UK. The research doctors looking after you will not be paid for including you in this study.

Who has reviewed the study?
All research in the NHS is looked at by an independent group of people, called a Research Ethics Committee to protect your safety, rights, wellbeing and dignity. This study has been reviewed and given favourable opinion by the North West – Preston Research Ethics Committee.

This completes Part 2 of the information sheet.

Thank you for taking the time to read about this study. A copy of this form and signed consent form will be provided for you to keep. Please contact any of the team members below should you require any further information or have any questions about the study.

Contact Details:

1) General information about research – please visit the NHS website: http://www.nhs.uk/Conditions/Clinical-trials/Pages/Introduction.aspx or Cancer Research UK website: http://www.cancerresearchuk.org/about-cancer/trials/types-of-trials/what-clinical-trials-are. General information about genetic testing can be found at http://www.nhs.uk/Conditions/Genetics/Pages/genetic-testing-and-counselling.aspx (although this website does not specifically provide information about testing genetics of cancer)

2) To obtain more information about this study, ask a question about any of the study procedures, for advice on whether you should participate or if you want to withdraw from the study, please contact one of the principal investigators or study co-ordinator:

   Dr Matthew Krebs, Consultant in Medical Oncology
   The Christie Experimental Cancer Medicine Unit, The Christie NHS Foundation Trust, Wilmslow Road, Manchester, M20 4BX. Tel: 0161 918 7672

   Dr Emma Dean, Consultant in Medical Oncology
   The Christie Experimental Cancer Medicine Unit, The Christie NHS Foundation Trust, Wilmslow Road, Manchester, M20 4BX. Tel: 0161 918 7672

   Ms Carla Siswick, Study Co-ordinator
   The Christie Experimental Cancer Medicine Unit, The Christie NHS Foundation Trust, Wilmslow Road, Manchester, M20 4BX. Tel: 0161 918 7699

3) If you are unhappy about any aspect of the study or wish to make a complaint, please contact the Chief Investigator of the study:

   Professor Andrew Hughes, Professor of Experimental Cancer Medicine
   The Christie Experimental Cancer Medicine Unit, The Christie NHS Foundation Trust, Wilmslow Road, Manchester, M20 4BX. Tel: 0161 918 7672

   Alternatively, you can submit a complaint directly to the Christie Patient Advice and Liaison Service (PALS) by calling 0161 446 8217 or emailing pals@christie.nhs.uk

The Christie NHS
NHS Foundation Trust

We Care, We Discover, We Teach

Patient ID: _______________

CONSENT FORM

Title: **TUMOUR CHARACTERISATION TO GUIDE EXPERIMENTAL TARGETED THERAPY – THE 'TARGET' STUDY**

1. I confirm that I have read and understood the information sheet dated 9th Feb 2015 version 2.0 for the above study. I have had the opportunity to consider the information, ask questions and have had these answered satisfactorily ☐

2. I understand that my participation is voluntary and that I am free to withdraw at any time without giving any reason and without my medical care or legal rights being affected ☐

3. I understand that relevant sections of my medical notes and data collected from the study may be looked at by regulatory authorities or by persons from the Trust where it is relevant to my taking part in this research. I give permission for these individuals to have access to this information. ☐

4. I agree to my GP being informed of my participation in the study ☐

5. I agree to provide my archival tumour specimen(s) and blood samples (as detailed in the information sheet) for genetic testing, which will include analysis of my normal (germline) DNA. I understand I will be given results about any genetic abnormalities that have direct relevance for the management of my cancer. ☐

What choices do I have for receiving results that do not have direct impact on the care of my current cancer?

I do not want to be told about results that may have significance for my biological family members ☐

I do not want to be told about results that are not related to my cancer but may have potential medical impact for me ☐

6. I agree that if I need any future operation or biopsy for clinically indicated reasons that any leftover samples, that are surplus to diagnostic need, may be used for genetic analysis and research purposes. ☐

7. I understand this research may include a range of tests (other than genetic analysis) on my donated samples if relevant to the study. ☐

Optional:

8. I agree to undertake a fresh tumour biopsy at the start of the study ☐

9. I agree to undertake a fresh tumour biopsy during the study and/or when my cancer stops responding to treatment if I am subsequently enrolled onto a clinical study to receive an experimental medicine designed to 'target' specific genetic faults identified in my tumour ☐

10. I agree my samples can be used to grow cancer cells in the laboratory including the use of animal (mice) experiments ☐

11. I agree to provide urine samples and/or hair follicles on up to 6 occasions ☐

12. At the end of the study I agree that my samples can be gifted to MCRC Biobank for future research according to their strict policies and ethical approvals. I understand future uses may include genetic or pharmaceutical research and my samples and anonymised data may be shared with collaborators across Europe and the World where data protection policies may be less stringent than the UK. No identifiable information such as name or contact details will be provided to these partners ☐

Signatures

Name of Patient<br>(BLOCK CAPITALS)	Date	Signature
Name of Researcher	Date	Signature
